# Effectiveness of antimicrobial prophylaxis at 30 versus 60 min before cesarean delivery

**DOI:** 10.1038/s41598-021-87846-z

**Published:** 2021-04-16

**Authors:** Hadas Rubin, Eyal Rom, Malak Wattad, Khadeje Seh, Natanel Levy, Ayellet Jehassi, Shabtai Romano, Raed Salim

**Affiliations:** 1 Department of Obstetrics and Gynecology, Emek Medical Center, 18101 Afula, Israel; 2 Department of Anesthesiology, Emek Medical Center, Afula, Israel; 3 Rappaport Faculty of Medicine, Technion, Haifa, Israel; 4 Biostatistics Unit, Emek Medical Center, Afula, Israel

**Keywords:** Health care, Medical research

## Abstract

This study aimed to examine the effect of antibiotic prophylaxis (AP) given within 30 compared to 30–60 min before skin incision on the incidence of infectious morbidity after cesarean delivery (CD). A retrospective cohort study was conducted at a single institution on data between 2014 and 2018. Women who delivered by CD were divided into two groups according to AP timing before skin incision: group 1 within 30 min, and group 2 from 30 to 60 min. The primary outcome was the incidence of any infectious morbidity. Overall, 2989 women were eligible: 2791 in group 1 and 198 in group 2. The primary composite outcome occurred in 125 women (4.48%) in group 1 and 8 women (4.04%) in group 2 (OR, 1.11; 95% CI 0.54–2.31; *P* = 0.77). The rate of surgical site infection only, was 1.08% in group 1 and 0.51% in group 2 (OR, 2.13; 95% CI 0.29–15.70; *P* = 0.72). The incidence was comparable between the groups in a separate sub-analysis restricted to laboring CDs and obese women. The rate of infectious morbidity was similar among women who received AP within 30 min and from 30 to 60 min before skin incision.

## Introduction

A major risk factor for postpartum infection is cesarean delivery (CD)^1^. The impact is substantial because the CD rate has considerably increased in the past two decades, and in several countries, nearly 1 in 3 pregnant women are delivered by CD^2,3^. Maternal infection is associated with increased perioperative mortality and morbidity that may lead to an increase in readmissions, prolonged hospital stays, and health care costs^4,5^.

Several studies from various populations have shown that nearly 3–12% of all CDs were complicated by surgical site infection (SSI)^1,2,6,7^. The favorable effect of antibiotic prophylaxis in reducing the occurrence of infectious morbidity related to CD, whether elective or emergency is also well established^8^.

The efficiency of antibiotic prophylaxis depends on their presence in sufficient concentrations throughout the operative period^9^. A number of meta-analysis concluded that antibiotic administration up to 60 min before skin incision, compared to after cord clamping, reduces the infection rate significantly^10–12^. Likewise, administering antibiotic prophylaxis more than 1 h before incision in CDs was associated with double the rate of SSI compared to 1 h before incision^13^.

Within the range of 1 h before incision, administering antimicrobial prophylaxis as close as possible to the incision time may not suffice to guarantee appropriate antimicrobial levels in tissue at the surgical site^14^. On the other hand, an increased interval may not compensate for the accelerated elimination present in pregnancy^15,16^. The present study aimed to examine the effect of antibiotic prophylaxis timing (up to 30 min vs. 30–60 min before surgery) on the rate of infectious morbidity.

## Results

A total of 3202 cesarean deliveries took place during the study period. Of those, 213 (6.7%) had missing data regarding the exact timing of antibiotic administration or had received prophylaxis outside the range of 0–60 min before incision and therefore were not included in the analysis. Overall, 2989 women were eligible and included in the final analysis: 2791 in group 1 and 198 in group 2.

Maternal demographics and obstetric characteristics are presented in Table 1. Intrapartum comparisons between the groups are presented in Table 2. The mean time from antibiotic prophylaxis to skin incision was 14.37 ± 7.92 min in group 1 and 37.62 ± 6.85 min in group 2 (*P* < 0.001; Table 3). The primary composite outcome occurred in 125 women (4.48%) who received antibiotic prophylaxis within 30 min before surgical incision and in 8 (4.04%) who received antibiotic prophylaxis 30–60 min before surgical incision (OR, 1.11; 95% CI 0.54–2.31; *P* = 0.77; Table 3). The incidence of SSI only was 1.08% and 0.51% among groups 1 and 2, respectively (OR, 2.13; 95% CI 0.29–15.70; *P* = 0.72). The difference between the groups did not differ significantly when any of the SSIs were tested separately (Table 3). The differences in the rates of the primary outcome and SSI between the groups did not differ significantly even after excluding women who underwent cesarean hysterectomy or women who received antibiotics up to 1 week before surgery for other medical reasons including prelabor rupture of membranes or pyelonephritis (data are not shown).

**Table 1:** Demographic and obstetric variables of women who received prophylactic antibiotic within 30 compared with 30–60 min before a skin incision.

Variables	Group 1: < 30 min (N = 2791)	Group 2: 30–60 min (N = 198)	*P*-value	OR (95% CI)
Maternal age, years	32.01 ± 5.70 (32.00)	32.67 ± 6.12 (32.50)	0.17	***
Maternal age > 35 years	809 (28.99)	70 (35.35)	0.06	0.75 (0.55–1.01)
Pregestational BMI (kg/m^2^)	25.77 ± 5.46 (24.67)	27.41 ± 6.46 (26.44)	0.0004	***
Pregestational BMI > 30 (kg/m^2^)	511 (18.31)	60 (30.30)	< 0.0001	1.94 (1.41–2.67)
Parity	2.48 ± 1.38 (2.00)	2.86 ± 1.68 (3.00)	0.004	***
Primiparous (1st birth)	801 (28.70)	47 (23.74)	0.13	1.29 (0.92–1.81)
Smoking	123 (4.41)	7 (3.54)	0.56	1.26 (0.58–2.73)
Twins gestations	245 (8.78)	20 (10.10)	0.53	1.17 (0.72–1.89)
Triplets	4 (0.14)	0.0 (0.0)	0.99	1.56 (0.08–29.08)
Any hypertension	102 (3.65)	7 (3.54)	0.93	1.04 (0.48–2.26)
Any diabetes	119 (4.26)	22 (11.11)	< 0.0001	0.36 (0.22–0.58)
Thrombophilia	79 (2.83)	7 (3.54)	0.57	0.80 (0.36–1.75)
Placental abruption	118 (4.23)	5 (2.53)	0.24	1.70 (0.69–4.22)
Placenta previa or low lying	67 (2.40)	5 (2.53)	0.81	0.95 (0.38–2.38)
Other maternal diseases	60 (2.15)	7 (3.54)	0.21	0.60 (0.27–1.33)
Previous one cesarean delivery or more	1323 (47.40)	114 (57.58)	0.006	0.66 (0.50–0.89)
Previous 3 cesarean delivery or more	686 (26.57)	74 (37.37)	0.0002	0.57 (0.42–0.77)

Data are mean ± standard deviation (median) or N (%) unless otherwise specified. Other maternal diseases: asthma; epilepsy; gastrointestinal disease; autoimmune disease; cardiac disease; thyroid disease; proteinuria.

**Table 2:** Peripartum variables of women who received prophylactic antibiotic within 30 compared with 30–60 min before skin incision.

Variables	Group 1: < 30 min (N = 2791)	Group 2: 30–60 min (N = 198)	*P*-value	OR (95% CI)
Gestational age at delivery (weeks)	37.9 ± 2.48 (38.30)	37.72 ± 1.82 (38.20)	0.009	***
Gestational age at delivery < 37 weeks	536 (19.21)	47 (23.74)	0.12	0.76 (0.54–1.07)
Induction of labor	298 (10.68)	10 (5.05)	0.01	2.25 (1.18–4.29)
Rupture of membranes	647 (23.18)	28 (14.14)	0.003	1.83 (1.22–2.76)
Any antibiotic within one week before delivery	224 (8.03)	20 (10.10)	0.30	0.78 (0.48–1.26)
Epidural use	268 (9.60)	11 (5.56)	0.06	0.55 (0.30–1.03)
Intrapartum fever	24 (0.86)	2 (1.01)	0.69	0.85 (0.20–3.62)
Chorioamnionitis	11 (0.39)	0.0 (0.0)	0.99	1.64 (0.01–27.97)
Type of cesarean			< 0.0001	
Planned	1417 (50.77)	127 (64.14)		1.00 (ref)
Unplanned	741 (26.55)	55 (27.78)		0.83 (0.60–1.15)
Laboring	633 (22.68)	16 (8.08)		0.28 (0.17–0.48)
Type of uterine incision			0.006	
Low transverse	2734 (97.95)	190 (95.96)		0.09 (0.02–0.33)
Corporal	51 (1.82)	4 (2.02)		0.10 (0.02–0.54)
Hysterectomy	6 (0.21)	4 (2.02)		1.00 (ref)
Bladder injury	9 (0.32)	2 (1.01)	0.16	0.32 (0.07–1.48)
Surgical time	40.13 ± 16.73 (36.95)	42.68 ± 19.92 (37.72)	0.21	***
Estimated blood lose, mL	609.61 ± 227.82 (500)	648.66 ± 481.05 (500)	0.32	***
Sterilization			0.10	
Bilateral tubal ligation	229 (8.20)	23 (11.62)		1.48 (0.94–2.34)
Bilateral salpingectomy	8 (0.29)	2 (1.01)		3.69 (0.78–17.51)
Type of scar closure			0.004	
Staples	1640 (58.76)	103 (52.02)		1.00 (ref)
Intracuticular	901 (32.28)	86 (43.43)		1.52 (1.13–2.05)
Glue	32 (1.15)	2 (1.01)		1.00 (0.24–4.21)
Missing data	218 (7.81)	7 (3.54)		0.51 (0.23–1.11)
Postpartum Hemoglobin < 8.0 g/dL	46 (1.64)	0.0 (0.0)	0.07	6.80 (0.42–110.87)
Blood transfusion	6 (0.21)	2 (1.01)	0.09	4.74 (0.95–23.62)
Difference between pre and postpartum hemoglobin, g/dL	0.52 ± 1.30 (0.40)	0.55 ± 1.43 (0.50)	0.94	***
White blood cell count before the cesarean	11.27 ± 3.81 (10.55)	10.64 ± 4.00 (9.92)	0.01	***
White blood cells count before the cesarean > 15.0 K/mcL	396 (14.19)	20 (10.10)	0.11	1.46 (0.91–2.35)
White blood cell count after the cesarean K/mcL	12.43 ± 3.66 (11.92)	12.04 ± 3.85 (11.21)	0.07	***
White blood cell count after the cesarean > 15.0 K/mcL	565 (20.24)	33 (16.67)	0.22	1.27 (0.87–1.87)
Neonatal birthweight, g	3120.59 ± 655.20 (3160)	3162.53 ± 618.61 (3135)	0.94	***
Apgar score at 5 min < 7	22 (0.79)	4 (2.02)	0.09	0.38 (0.13–1.11)

Data are mean ± standard deviation (median) or N (%) unless otherwise specified.

**Table 3:** Outcomes of women who received prophylactic antibiotic within 30 compared with 30–60 min before skin incision.

Outcomes	Group 1: < 30 min (N = 2791)	Group 2: 30–60 min (N = 198)	*P*-value	OR (95% CI)
Time from prophylactic antibiotic to skin incision, min	14.37 ± 7.92 (14.82)	37.62 ± 6.85 (35.33)	< 0.001	***
Composite outcome (any infection)	125 (4.48)	8 (4.04)	0.77	1.11 (0.54–2.31)
Surgical site infection	30 (1.08)	1 (0.51)	0.72	2.13 (0.29–15.70)
Superficial scar infection	21 (0.75)	0.0 (0.0)	0.39	3.09 (0.19–51.25)
Deep scar infection	1 (0.04)	0.0 (0.0)	0.99	4.67 (0.19–114.95)
Deep organ infection	8 (0.29)	1 (0.51)	0.46	1.76 (0.22–14.13)
Urinary tract infection	42 (1.50)	5 (2.53)	0.24	1.70 (0.66–4.34)
Pneumonia	55 (1.97)	2 (1.01)	0.59	0.51 (0.12–2.10)
Length of hospitalization after surgery	4.11 ± 1.41 (3.93)	4.24 ± 2.10 (3.99)	0.84	***

Data are mean ± standard deviation (median) or N (%) unless otherwise specified.

A regression model was performed to adjust for antepartum and intrapartum variables that differed significantly between the groups. The results showed that the rates of the primary composite outcome and SSI were still comparable between the groups (adjusted OR, 0.98; 95% CI 0.46–2.07; *P* = 0.95; adjusted OR, 0.61; 95% CI 0.08–4.64; *P* = 0.64, respectively).

In further analysis, we increased the timing categories to 4 (0–15, 16–30, 31–45, and 46–60 min) before skin incision. The rates of the primary outcome did not differ.

A separate subanalysis was performed regarding laboring CDs and CDs in women with a BMI > 30 kg/m^2^ or > 35 kg/m^2^. Again, the rates of the primary outcome and SSI did not differ between the groups (Table 4).

**Table 4:** Infectious morbidity in obese women and intrapartum cesareans according to timing of antibiotic prophylaxis before skin incision.

Outcomes	Group 1: < 30 min	Group 2: 30–60 min	*P*-value	OR (95% CI)
	Pregestational BMI > 30 kg/m^2^ (N = 511)	Pregestational BMI < 30 kg/m^2^ (N = 60)		
Composite outcome (any infection)	25 (4.89)	1 (1.67)	0.51	3.04 (0.40–22.81)
Surgical site infection	7 (1.37)	0 (0.0)	0.99	1.80 (0.10–31.89)
Urinary tract infection	6 (1.17)	1 (1.67)	0.54	1.43 (0.17–12.05)
Pneumonia	12 (2.35)	0 (0.0)	0.63	0.33 (0.02–5.65)

	Pregestational BMI > 35 kg/m^2^ (N = 173)	Pregestational BMI < 35 kg/m^2^ (N = 24)		
Composite outcome (any infection)	4 (2.31)	0 (0.0)	0.99	1.30 (0.07–24.91)
Surgical site infection	1 (0.58)	0 (0.0)	0.99	0.43 (0.02–10.76)
Urinary tract infection	1 (0.58)	0 (0.0)	0.99	2.35 (0.09–59.24)
Pneumonia	2 (1.16)	0 (0.0)	0.99	1.40 (0.07–30.03)

	Laboring cesarean (N = 633)	Laboring cesarean (N = 16)		
Composite outcome (any infection)	43 (6.79)	1 (6.25)	0.99	1.09 (0.14–8.47)
Surgical site infection	13 (2.05)	0 (0.0)	0.99	0.72 (0.04–12.62)
Urinary tract infection	18 (2.84)	1 (6.25)	0.38	2.28 (0.29–18.19)
Pneumonia	12 (1.9)	0 (0.0)	0.99	1.51 (0.09–26.54)

Data are mean ± standard deviation (median) or N (%) unless otherwise specified.

Finally, a univariate logistic regression model was performed to assess differences in the relative frequency of infections as a function of time from antibiotic administration to skin incision. The results showed that for every increase of 1 min between antibiotic administration and skin incision, the incidence of the primary outcome decreased by a factor of 1.02 (OR, 0.98; 95% CI 0.96–0.99; *P* = 0.018). The AUC was 0.55 (95% CI 0.50–0.61). We then strived to find the best logistic classifier, namely, the most influential factors that can predict the risk of infection. To this end, we used multivariable logistic regression. The results showed that for every 1 min increase between antibiotic administration and skin incision, the incidence of the primary outcome decreased by a factor of 1.02 (adjusted OR, 0.98; 95% CI 0.96–0.99; *P* = 0.021); however, there was a significant increase in the AUC to 0.62 (95% CI 0.57–0.67). This model was adjusted to the type of incision closure. Performing the same analysis among women with laboring CD or with BMI > 30 kg/m^2^ did not show a significant trend.

## Discussion

The overall infectious morbidity found in the current study is comparable to other reports where antibiotic prophylaxis was given before skin incision^17^. The results show that the difference in post-cesarean infectious morbidity was not affected whether antibiotic prophylaxis was given within 30 min or from 30 to 60 min before skin incision. The SSI rate alone was also not affected. The results did not differ when the analysis was performed on medical data for obese women only or when the analysis was restricted to laboring CDs only. Although a significant trend (decrease) in the incidence of infectious morbidity was found as the time between antibiotic prophylaxis administration (within the range of 60 min) and the skin incision was increased, the AUC was relatively low.

The use of a retrospective database is one of the limitations of the current study. Nevertheless, inaccuracies were minimized by the use of multiple sources and by manual validation of individual medical files. Furthermore, the groups were unbalanced, with fewer women in the 30–60 group. Additionally, the fact that the study was conducted at a single hospital may limit its generalizability. The major strength of this study was the inclusion of a large sample size. Additionally, the use of standardized guidelines and surgical techniques is probably a distinctive advantage related to a single-center study.

The effect of antibiotic prophylaxis prior to skin incision in reducing infectious morbidity, compared to after cord clamping, is well established^8–12,12,13,13–20^. The American College of Obstetricians and Gynecologists recommended antimicrobial prophylaxis for all CDs and that prevention has to be administered within 60 min prior to skin incision^8^. Nevertheless, this recommendation does not describe a definitive point in time at which antibiotic prophylaxis should be provided within this window in order to obtain adequate tissue levels for prevention.

The objective of preoperative antibiotic prophylaxis is to reduce postoperative infection, presumably by delivering adequate antibiotics to the surgical site to suppress bacterial growth^21^. Elkomy et al. showed that if the time of antibiotic prophylaxis administration is close to 1 h before incision, the minimum inhibitory concentration (MIC) in maternal blood is reduced at surgery compared to an administration less than 30 min before incision^15^. Hence, it is necessary to shorten the dosing interval or increase the dose in pregnancy to compensate for accelerated elimination and preserve a free drug plasma concentration similar to that in nonpregnant adults^15,17^.

A randomized trial evaluated the administration of cefazolin at the time of skin incision (at-incision group) compared with administration after umbilical cord clamping (cord-clamping group) in laboring CDs. The results showed a significant decrease in the incidence of endometritis but not in wound infection among the at-incision group^22^. The lack of protective effect on wound infection may be related to the timing of antibiotic administration, implying that administration at incision may still not differ significantly from administration at cord clamping.

Data is lacking regarding a definitive point in time at which antibiotic prophylaxis should be provided within 1 h before skin incision in cases of CDs to attain adequate tissue levels for prevention. Data regarding other surgical procedures is conflicting. A prospective observational cohort study analyzed the incidence of SSI by the timing of antimicrobial prophylaxis in a series of 3836 surgical procedures other than CDs. When antibiotic prophylaxis was used 30–59 min before incision, less SSI was observed compared to the administration at less than 30 min. The authors concluded that antibiotic prophylaxis timing should ensure that serum and tissue drug levels at the beginning of the operation exceed the MIC for organisms likely to be present in the surgical environment. Administering surgical antimicrobial prophylaxis close to the incision time may not suffice to guarantee appropriate tissue levels at the surgical site^14^. In contrast, 29 hospitals prospectively obtained information from 4472 randomly selected surgical procedures other than CDs. The SSI risk increased incrementally as the interval of time between antibiotic infusion and the incision increased, with a trend toward lower risk occurring when antibiotic prophylaxis was given within 30 min prior to incision^23^. Compared to other surgical procedures presented in the previous reports^14,23^, the incidence of SSI in CDs did not differ whether antibiotic prophylaxis was given within 30 min or from 30 to 60 min of the incision, according to the present study results.

In conclusion, implementing a refined optimal time window for the prophylactic administration of antibiotics in clinical practice and adhering to an optimal time window are probably hard. Nevertheless, the aim should be to apply prophylaxis at the optimal time, despite practical and logistic difficulties. Based on the current study results, the rate of infectious morbidity was comparable when antibiotic prophylaxis was given within 30 min or between 30 and 60 min before incision. Compared to other nonobstetric surgical procedures where the optimal window was within 30 min, the results of the current study may ease the adherence and lead to a higher compliance rate.

## Material and methods

We conducted a retrospective cohort study at a single university teaching institution with approximately 4400 annual deliveries. Institutional review board (IRB) at Emek Medical Center, Afula, affiliated to the Rappaport Faculty of Medicine, Technion, Haifa, Israel, approved the study protocol. All methods were carried out in accordance with relevant guidelines and regulations. Informed consent was waived by the IRB at Emek Medical Center, the largest medical center in the North—East of Israel, because of the study’s retrospective nature. The study consisted of all women who underwent cesarean delivery at a gestational age of 24 weeks or more between January 2014 and March 2018. Women who received antibiotic prophylaxis after skin incision due to the urgency of the procedure or had missing data regarding the exact timing of prophylaxis administration were excluded.

Women were identified by using the computerized labor charts and electronic medical records at admission and discharge. Data were extracted from labor, anesthesia, and postpartum hospitalization charts as well as computerized outpatient diagnosis and clinic records, including visits to a physician, laboratory results, and visits to any gynecologic emergency unit up to 3 months after discharge. The computerized system in hospitals and outpatient clinics in Israel is a shared, secure, web-based system that allows the medical staff in a hospital to view outpatients’ data and visits and vice versa. Trained research staff members ascertained all medical files of individual cases manually for validation.

SSI was defined based on the US Centers for Disease Control and Prevention criteria^18,19^. Women who had superficial incisional infection, deep incisional infection or endometritis were categorized as having SSI. Endometritis was defined as the presence of temperature ≥ 38 °C on two separate occasions, or temperature ≥ 39 °C and at least 1 of the following signs with no other recognized cause: abdominal pain, uterine tenderness, or purulent discharge. Incisional SSI was defined as superficial or deep, characterized by cellulitis or erythema and localized swelling or purulent discharge from the incision site regardless of fever. Wound seroma, hematoma, or separation alone was not considered as an infection. Diagnosis of abdominal or pelvic abscess necessitated radiologic or surgical confirmation. Other infections included urinary tract infection with the presence of culture-positive urine and pneumonia in the presence of clinical and radiological confirmation.

Each CD was categorized as planned, unplanned but not in labor and with intact membranes or laboring CD. The cesarean surgical approach was standardized during the study period.

All participants received chlorhexidine skin preparation unless there was a documented allergy, in which case, povidone-iodine was used. Standard sterile draping of participants was performed. Intravenous 1 g cefazolin was administered prior to skin incision. In women with a body mass index (BMI) of 35 kg/m^2^ or more, 2 g was given. Clindamycin was given in cases of allergy to cefazolin. A Pfannenstiel incision was the preferred skin incision. Surgical dressings were removed within 24 h postoperatively^24^. Women were examined daily in the hospital by a physician and the registered nurses.

Women were divided according to the time of antimicrobial prophylaxis administration before skin incision, into two groups: group 1 received prophylaxis up to 30 min before skin incision and group two from 30 to 60 min before skin incision.

The primary outcome was a composite of SSI (endometritis, wound infection) or other infections, including abdominal pelvic abscess, urinary tract infection, or pneumonia occurring up to 3 months after surgery.

We assumed that antimicrobial prophylaxis given 30–60 min before incision would result in more effective concentrations throughout the operative period and may lead to a reduced infection rate compared to antimicrobial prophylaxis given up to 30 min before skin incision. Accordingly, in order to detect a decrease in the rate of the primary outcome from 5% observed previously at our institution^20^ to 3% between the groups, 3200 women were needed to achieve 80% power with α = 0.05. Based on the sample size calculation, the retrospective data collection began in 2014 and ended in 2018 to obtain an appropriate sample size.

### Statistical analysis

To analyze the differences between the two groups as a function of the administering time of the antimicrobial prophylaxis before skin incision (up to 30 min vs. 30–60 min) adjusted to the selected set of categorical variables, a series of ${\chi }^{2}$ tests or Fisher exact tests (once the assumptions of the parametric ${\chi }^{2}$ test were not met) were conducted. The odds ratio (OR) with its corresponding 95% confidence interval (CI) was computed. For the empty cells in the contingency tables, a factor of 0.5 was added to each cell before computing the OR and its corresponding 95% CI. In order to test whether the groups differed in the continuous outcomes, a Student *t*-test or the nonparametric Mann–Whitney U test (if the sample means had not satisfied the normality assumption) was conducted. Using the variables that were significant in the univariate analysis, multivariate logistic regression was estimated. From the results of this model, OR was adjusted, and its corresponding 95% CI was obtained.

To analyze the relationship between the administering time of the antibiotic prophylaxis and the primary outcome, two separate analyses adjusted to two different divisions of the time interval were conducted. In the first analysis, the timeline was divided into the following four disjoint time intervals: 0–15 min, 16–30 min, 31–45 min, and 46–60 min. In the second analysis, the timeline was considered as a continuous variable. For both parts, we estimated univariate and multivariate logistic regression models, respectively. For the last analysis, the inclusion criteria of variables in the regression model were based on the variables’ potential role as primary outcome risk factors. Due to a large number of candidate explanatory variables, the variable selection was performed by the forward selection method. The inclusion criterion was a significant increase in the area under the receiver operating characteristic curve (AUC)^25^. The AUC statistic provides an indication of the classifier’s efficacy. Once the increase in the AUC was insignificant, we stopped the process and took the model from the previous step as the final one. For each of the regression coefficients, the 2-tailed *P* values, where *P* < 0.05 was considered statistically significant, were computed.

Statistical analyses were performed using SAS software (SAS Institute, 2013. SAS/STAT, ver. 9.4. SAS Institute Inc., Cary, NC, USA, https://www.sas.com) and R 3.6.1 (R Core Team, 2018, https://www.R-project.org/).

## Data Availability

The datasets generated and analyzed during the current study are not publicly available since this is a retrospective study and the data was extracted from patient electronic files. Anonymized participant data will be made available upon reasonable request directed to the corresponding author. Requests will be reviewed and approved by the corresponding author and collaborators on the basis of scientific merit.
